# Omics Approach to Axonal Dysfunction of Motor Neurons in Amyotrophic Lateral Sclerosis (ALS)

**DOI:** 10.3389/fnins.2020.00194

**Published:** 2020-03-25

**Authors:** Naoki Suzuki, Tetsuya Akiyama, Hitoshi Warita, Masashi Aoki

**Affiliations:** ^1^Department of Neurology, Tohoku University School of Medicine, Sendai, Japan; ^2^Department of Neurology, Shodo-kai Southern Tohoku General Hospital, Miyagi, Japan

**Keywords:** amyotrophic lateral sclerosis (ALS), omics analysis, axonal dysfunction, local translation, axon branching, motor nerve organoid, human induced pluripotent stem cell (hiPSC)-derived motor neuron

## Abstract

Amyotrophic lateral sclerosis (ALS) is an intractable adult-onset neurodegenerative disease that leads to the loss of upper and lower motor neurons (MNs). The long axons of MNs become damaged during the early stages of ALS. Genetic and pathological analyses of ALS patients have revealed dysfunction in the MN axon homeostasis. However, the molecular pathomechanism for the degeneration of axons in ALS has not been fully elucidated. This review provides an overview of the proposed axonal pathomechanisms in ALS, including those involving the neuronal cytoskeleton, cargo transport within axons, axonal energy supply, clearance of junk protein, neuromuscular junctions (NMJs), and aberrant axonal branching. To improve understanding of the global changes in axons, the review summarizes omics analyses of the axonal compartments of neurons *in vitro* and *in vivo*, including a motor nerve organoid approach that utilizes microfluidic devices developed by this research group. The review also discusses the relevance of intra-axonal transcription factors frequently identified in these omics analyses. Local axonal translation and the relationship among these pathomechanisms should be pursued further. The development of novel strategies to analyze axon fractions provides a new approach to establishing a detailed understanding of resilience of long MN and MN pathology in ALS.

## Introduction

Amyotrophic lateral sclerosis (ALS) is a devastating adult-onset neurodegenerative disorder (Brown and Al-Chalabi, 2017). Both the upper and lower motor neurons (MNs) are affected, such that the disorder is characterized by muscle weakness with spasticity and atrophy. Approximately 10% of ALS occurrence is familial (Ghasemi and Brown, 2018). Since the identification in 1993 (Rosen et al., 1993) of *copper/zinc superoxide dismutase 1* (*SOD1*) in ALS patients with an autosomal dominant trait in 1993 (Aoki et al., 1993), more than 25 genes have been reported as causative genes of familial ALS (Maday et al., 2014; De Vos and Hafezparast, 2017; Ghasemi and Brown, 2018; Cook and Petrucelli, 2019).

The pathomechanisms of ALS have been examined using familial ALS models. Intracellular generation of reactive oxygen species production (Borchelt et al., 1994; Wiedau-Pazos et al., 1996; Howland et al., 2002) and unfolded protein response/endoplasmic reticulum (ER) stress (Kikuchi et al., 2006; Kieran et al., 2007; Urushitani et al., 2008) have been inferred from the discovery of SOD1 as a factor. A cell non-autonomous effect (Boillee et al., 2006; Di Giorgio et al., 2007; Nagai et al., 2007; Yamanaka et al., 2008; de Boer et al., 2014) has also been examined in mutant *SOD1*-transgenic mouse and cellular models. Mutations in the RNA-binding protein (RBP) *TAR DNA-binding protein* (*TARDBP* encoding TDP-43) can result in ALS (Kabashi et al., 2008; Rutherford et al., 2008), and cytoplasmic TDP-43 inclusions have been reported in over 90% of cases of sporadic ALS (Mackenzie et al., 2010). In 2009, *fused in sarcoma* (*FUS*) was determined in 2009 to be the causative gene of ALS (Kwiatkowski et al., 2009; Vance et al., 2009). FUS and TDP-43 have similar structural characteristics, including an RNA recognition motif (RRM), a nuclear export signal (NES), a nuclear localization signal (NLS), and prion-like domains (PrLDs) (Kapeli et al., 2017). The C-terminal NLS site regulates the nucleocytoplasmic localization of FUS and is a hotspot for mutations in familial ALS (Kwiatkowski et al., 2009; Vance et al., 2009; Suzuki et al., 2010; Nishiyama et al., 2017). Abnormal phase separation of FUS is involved in this pathomechanism (Guo et al., 2018; Hofweber et al., 2018; Qamar et al., 2018). NLS mutations impair the nuclear import of FUS, and the level of mislocalized cytoplasmic FUS is correlated to the severity of the clinical ALS phenotypes (Dormann et al., 2010). In addition, recent reports have demonstrated that the abnormal NLS function results in the aberrant accumulation of mutant FUS in the cytoplasm (Ichiyanagi et al., 2016; Guo et al., 2018; Hofweber et al., 2018; Qamar et al., 2018; Yoshizawa et al., 2018). Previous studies have found that the toxic gain of function occurring with mutant *FUS* is crucial for neurodegeneration (Scekic-Zahirovic et al., 2016; Sharma et al., 2016; Shiihashi et al., 2016).

A hexanucleotide repeat expansion in *chromosome 9 open reading frame 72* (*C9orf72*) (DeJesus-Hernandez et al., 2011; Renton et al., 2011) is the most common cause of ALS when examined in Western countries (Balendra and Isaacs, 2018). Loss of function of C9ORF72 (Burberry et al., 2016; O’Rourke et al., 2016), toxic gain of function of C9ORF72 due to repeat RNA (Peters et al., 2015; Jiang et al., 2016), and toxic gain of function due to proteins with dipeptide repeats resulting from repeat-associated non-ATG translation (Mori et al., 2013; Kwon et al., 2014; Mizielinska et al., 2014; Wen et al., 2014; Chew et al., 2015) have been suggested as disease mechanisms.

These findings are mainly focused on the event in the cytoplasm of MNs. Actually, long axons, which have lengths of up to 100 cm in humans, are characteristic of MN morphology, and connect the soma of MNs to the skeletal muscles. In ALS, MNs are dysfunctional due to axonal degeneration (Ferraiuolo et al., 2011), that occurs prior to the motor phenotype in ALS (Fischer et al., 2004; Roy et al., 2005). Consistent with this observation, transgenic models of ALS also demonstrate abnormal axons and other degenerative processes, followed by the death of MNs (Armstrong and Drapeau, 2013; Tian et al., 2016; Fujimori, 2018). Other studies have revealed that axonal damage occurs earlier than the death of cell bodies and subsequent symptoms in patients; such symptoms become apparent only after the loss of many MNs (Dadon-Nachum et al., 2011).

Various reviews have described the physiological and pathological features of neuronal axons, including cargo transport within axons, local translation, and the axonal transcriptome (Jung et al., 2012; Maday et al., 2014; Batista and Hengst, 2016; Neto et al., 2016; Brady and Morfini, 2017; De Vos and Hafezparast, 2017). However, because primary neurons from patients cannot be easily obtained and because axons produce low sample yields and are difficult to culture, the details of the pathological mechanisms of ALS remain unclear. To further elucidate the resilience and pathomechanisms in MN axons, this review summarizes omics analyses of the axon compartment using microfluidic devices and *ex vivo* samples. Intra-axonal transcription and local axonal translation are the mechanisms of ALS emerging in the field, as discussed in the following.

## Accumulating Evidence of Axonal Dysfunction in ALS

**FIGURE 1:**
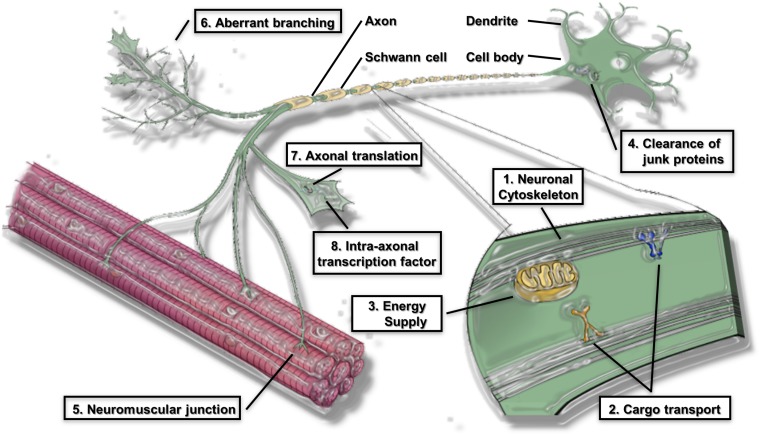
Proposed pathomechanism of ALS in axon compartments. (1) Neuronal cytoskeleton; (2) cargo transport within axons; (3) axonal energy supply; (4) clearance of junk protein; (5) Neuromuscular junction (NMJs); (6) aberrant axonal branching; (7) Axonal translation; (8) Intra-axonal transcription factors are prominent features of the proposed pathomechanism.

The global pathomechanisms of axons in ALS are considered next, in an overview of the current knowledge of axonal events in MNs. This section classifies the pathomechanisms of axonal dysfunction into six subsections, including neuronal cytoskeleton, cargo transport within axons, axonal energy supply, clearance of junk protein, neuromuscular junctions (NMJs), and aberrant axonal branching (Figure 1). As mentioned in the introduction, an increasing number of genes have been found as causative or associated genes for ALS. Evidence of axon pathomechanisms from the genetics of ALS is also accumulating (Table 1). These mechanisms are explained in each subsection.

**TABLE 1:** Motor neuron disease-associated mutations and axonal pathology.

Disease	Gene	Protein	Axonal pathology
ALS1	SOD1	Superoxide dismutase 1	Impaired transport of mitochondria, microtubule stability, modulation of motor proteins via p38 MAP kinase etc.
ALS2	ALS2	Alsin	Impaired endocytic trafficking, signaling endosomes
ALS5/SPG11	SPG11	Spatacsin	Axonal destabilization, reduced tubulin acetylation, reduced anterograde vesicle transport
ALS6	FUS	FUS	Defective transport of mitochondria, aberrant microtubule acetylation, NMJ deformity, aberrant axon branching, Fos-B overexpression
ALS8	VAPB	Vesicle-associated membrane protein-associated protein B	Impaired transport of mitochondria and vesicles
ALS10	TARDBP	TAR DNA-binding protein 43	Defective transport of mitochondria and mRNP granules; reduced expression of dynactin 1; aberrant microtubule stability/acetylation
ALS17	CHMP2B	Charged multivesicular body protein 2B	Impaired endocytic trafficking, signaling endosomes
ALS12	OPTN	optineurin	Progressive dysmyelination and axonal degeneration through engagement of necroptotic machinery in the CNS, including RIPK1
ALS18	PFN1	Profilin l	Decreased bound actin levels and can inhibit axon outgrowth. Primary motor neurons expressing mutant PFN1 display smaller
ALS22	TUBA4A	Tubulin, alpha 4a	Destabilization of microtubules, general transport defect
ALS23	ANXA11	annexin All	Molecular tether between lysosomes and RNA granules in axon
ALS25/SPGIO	KIF5A	Kinesin heavy chain	Reduced kinesin-1 mediated transport, impaired neurofilament transport
FALS/HMN7B/Perry syndrome	DCTN1	Dynactin 1 (p150, glued homolog, Drosophila)	Altered axonal transport and vesicle trafficking, impaired signaling endosome trafficking
FTDALS1	C9orf72	C90RF72	Defective transport of mitochondria
SPG4	SPAST	Spastin	Destabilization of microtubules, impaired transport of mitochondria and vesicles
SPG30	KIF1A	Kinesin Family Member 1A	Reduced kinesin-3 mediated transport
SBMA	AR	Androgen receptor	Defective retrograde and anterograde transport, modulation of motor proteins via JNK

*Modified from Maday et al. (2014); De Vos and Hafezparast (2017), and Ghasemi and Brown (2018). Also refer to OMIM (https://www.omim.org/) as of June 2019.*

### Neuronal Cytoskeleton

The axon can be visualized as a railway, and the electric signal should be transferred from one train terminal station (the cell body) to another terminal station (the skeletal muscle). Mutations in genes associated with microtubules have been identified as the causative events in ALS.

Several variants of the gene encoding α-tubulin, *TUBA4A*, destabilize the microtubule network and reduce the repolymerization capability of this network (Smith et al., 2014). A missense mutation in the *tubulin-specific chaperone E* gene, causing MN degeneration in the progressive motor neuronopathy model mouse, ends in microtubule and axonal defects similar to those induced by the ALS-linked *TUBA4A* variation in patients (Bommel et al., 2002; Martin et al., 2002).

Mutations in *profilin 1* (*PFN1*) can also lead to familial ALS. PFN1 converts monomeric actin to filamentous actin. Ubiquitinated aggregates are present in cells that express mutant *PFN1*, and many of these aggregates include TDP-43, which is associated with ALS (Wu et al., 2012). Such cells also have lower levels of bound actin and block axon growth. Primary MNs that harbor mutant *PFN1* have a lower ratio of monomeric to filamentous actin and smaller growth cones. The PFN1 transgenic mouse has also been observed to recapitulate the phenotype of MN disease (Fil et al., 2017).

NIMA (never in mitosis gene A)-related kinase 1 (NEK1) has been linked to cilia formation, microtubule stability, and neuronal morphology (Thiel et al., 2011). The *NEK1* gene was identified as a susceptibility factor for ALS (Brenner et al., 2016; Kenna et al., 2016). Using *in vivo* imaging, axonal degeneration was identified as an early event in the *SOD1* and *C9ORF72* repeat expansion mouse models of ALS (Tian et al., 2016). Neurofilament L transcripts are reduced in ALS (Bergeron et al., 1994). Neurofilaments are also found in a spheroid structure (large axonal swelling) (Corbo and Hays, 1992). Neurofilament light (NFL) and phosphorylated neurofilament heavy (pNFH) are also known as biomarkers for ALS (Brettschneider et al., 2006; Steinacker et al., 2016).

Thus, a dysfunctional cytoskeleton plays a role in ALS pathogenesis.

### Cargo Transport Within Axons

Maintenance of the function and structure of all types of cells in mammals requires the intracellular transport of cargo. This transport is especially important in neurons because of their axonal and cell body polarization (De Vos and Hafezparast, 2017). Proteins and mRNA, as well as organelles, are generally synthesized in the soma and transported along the axon. Proper transport is required for the distribution of this cargo at the right time and place in the axon. Using electron microscopy of autopsy samples from ALS cases, defects in the cargo transport within axon transport in ALS have been observed. The studies of this transport defect revealed that the proximal axons of large MNs harbor abnormal accumulation of mitochondria, phosphorylated neurofilaments, and lysosomes (Hirano et al., 1984a, b; Okada et al., 1995; Rouleau et al., 1996). In addition, spheroids present in the axons contain different types of vesicles, lysosomes, mitochondria, neurofilaments, and microtubules (De Vos and Hafezparast, 2017). The accumulation of phosphorylated neurofilaments at the initial segment of MN axons is a major pathological characteristic of ALS (Ackerley et al., 2004; Brady and Morfini, 2017).

Aberrant cargo transport within axons occurs early in ALS disease progression (Williamson and Cleveland, 1999; De Vos et al., 2007). For example, an altered transport of mitochondria in axons has been demonstrated in two different mutant *SOD1*-G93A transgenic mouse models of ALS (Magrane et al., 2014). The slow anterograde transport of cytoskeletal components is decreased during the months prior to the initial neurodegeneration in mutant *SOD1*-G37R transgenic mice, a change that has been exhibited using metabolic labeling studies (Williamson and Cleveland, 1999). In mutant *SOD1*-G93A transgenic mouse models, inhibiting p38 MAPK α rescues retrograde cargo transport defects within axons (Gibbs et al., 2018).

TDP-43 functions as an mRNA transporter across the axonal cytoskeleton, and ALS-related mutations in TDP-43 alter this transport function (Alami et al., 2014). Mitotracker and Lysotracker experiments in *FUS*-mutant iPSC-derived MNs have demonstrated that defects in the cargo transport within axons can be rescued by histone deacetylase 6 inhibition (Guo et al., 2017).

Mutations in the genes that code for the motor protein dynactin (*DCTN1*) (Puls et al., 2003) have been identified in the genetic analyses of familial ALS. Mutant dynactin binds weakly to microtubules, compared with the binding of wild-type proteins. ALS and slowly progressing, autosomal dominant, distal hereditary motor neuropathy in vocal paresis (HMN7B) are due to loss-of-function mutations in *DCTN1* (Puls et al., 2003; Munch et al., 2004; Yan et al., 2015). DCTN1 expression is also found to be downregulated in ALS-derived autopsy samples (Jiang et al., 2005).

*Kinesin family member 5A* (*KIF5A*) is a newly identified gene that plays a role in ALS (Brenner et al., 2018; Nicolas et al., 2018). Mutations that occur in the N-terminal motor domain of KIF5A cause an autosomal dominant type of hereditary spastic paraplegia known as spastic paraplegia (SPG)10, as well as Charcot–Marie–Tooth disease type 2 (Fichera et al., 2004). In contrast, mutations associated with ALS are mainly found in the C-terminal domain, which is important for binding cargo. Patients with loss-of-function *KIF5A* mutations have longer survival times than those with typical ALS (Brenner et al., 2018; Nicolas et al., 2018). Mutations in *KIF5A*, as well as *KIF1A*, which are loss-of-function mutations, are present in the motor or neck domains (Ebbing et al., 2008; Citterio et al., 2015).

In addition to MNs, mature sensory axons also possess a complex series of mRNA. A microtubule-stabilizing agent, paclitaxel, which impairs cargo transport within axons, results in sensory neuropathy (Gumy et al., 2011). Defects in the cargo transport within axons are common to various neurodegenerative diseases. Impaired cargo transport in axons can cause neurodegeneration (Millecamps and Julien, 2013).

### Axonal Energy Supply

The mitochondria play an important role in meeting the axonal energy demand as they generate ATP through oxidative phosphorylation (Chamberlain and Sheng, 2019). Following their synthesis in the cell body, the mitochondria enter the axon where they undergo robust trafficking and accumulate at the nodes of Ranvier to meet metabolic needs (Zhang et al., 2012). Disruption of the mitochondrial activity, transport proteins, and microtubule association likely leads to dysfunctional mitochondrial transport in neurodegenerative diseases. Energy deficits in injured axons are caused by damage to the mitochondria following damage to axons, a decrease in mitochondrial transport in axons of mature neurons, and an increased energy consumption (Zhou et al., 2016). During regeneration, the axons adapt to this increased energy demand by changing the dynamics of the mitochondria (Kiryu-Seo and Kiyama, 2019). Mutations in *RAPGEF2* mutations impair microtubule stability and the mitochondria distribution in axons (Heo et al., 2018). Reduction in mitochondrial Rho GTPase 1 (Miro 1), the outer mitochondrial membrane protein, leads to anterograde axonal transport defects (Moller et al., 2017). The imbalance between mitochondrial fission and fusion leads to abnormal mitochondrial morphology, underlies axonal damage, and is a potential therapeutic target for treating SPG15 and SPG48 (Denton et al., 2018). The ER and mitochondria form complex sites of interactions known as mitochondria-associated membranes (Gentile et al., 2019). Decreased ER-mitochondria association can occur as a result of loss-of-function mutations in *SIGMAR1*, leading to impaired retrograde transport and, ultimately, to axonal degeneration and MN death (Bernard-Marissal et al., 2015; Watanabe et al., 2016).

Astrocytes and oligodendrocytes may meet the axonal energy demand (Kang et al., 2010; Lee et al., 2012; Morrison et al., 2013). A deficiency in monocarboxylate transporter 1 (MCT1) was observed in oligodendroglia in the ventral cord of *SOD1* transgenic mice and in the motor cortex of ALS patients (Kang et al., 2013; Philips et al., 2013). The removal of the *SOD1* mutation from oligodendroglial precursor cells was observed to result in marked attenuation of the progression of the disease (Kang et al., 2013). Reducing the expression of *MCT1* in oligodendroglia is the pathomechanism involving the energy supply that contributes to MN degeneration in ALS.

### Clearance of Junk Protein

The ubiquitin proteasome and autophagy clearance systems are significant homeostatic processes engaged in eliminating defective organelles and aggregated proteins throughout the life span of the neuron. Impairment of the ubiquitin proteasome degradation system in MNs has been reported to replicate the ALS phenotype in mice (Tashiro et al., 2012). Mice with MN-specific, conditional knockout of the proteasome subunit *Rpt3* exhibit locomotor dysfunction, progressive MN loss, and gliosis (Tashiro et al., 2012). Constitutive autophagy in neurons also maintains cellular homeostasis by balancing the synthesis and degradation of proteins, particularly within the distal axonal processes (Maday and Holzbaur, 2016). Several genes, such as *valosin-containing protein*, are involved in the protein degradation process (Johnson et al., 2010).

What about in the axon fraction? Disruption of the endosomal-lysosomal system due to ALS2/Alsin loss exacerbates the phenotype of *SOD1*-H46R transgenic mice by accelerating the accumulation of misfolded proteins and immature vesicles in the spinal cord (Hadano et al., 2010). In the early symptomatic and even presymptomatic *SOD1*-H46R transgenic mice, degenerating and swollen spinal axons with the accumulation of autophagosome-like vesicles have been observed (Hadano et al., 2010). A recent study also reported impairment of the degradation of autophagic vacuoles that engulf damaged mitochondria from distal axons in the *SOD1*-G93A transgenic mouse model (Xie et al., 2015). The clearance of dysfunctional mitochondria from axons may be mediated by syntaphilin, a mitochondria-anchoring protein, which is expressed at high levels in the early disease stages of ALS in MNs (Lin et al., 2017). *FUS* mutation causes axonal retention of the FUS protein prior to its aggregation, which is caused by poly(ADP-ribose) polymerase-dependent DNA response signaling (Naumann et al., 2018). The authors of this review also observed the accumulation of mutant FUS protein in the neurites of *FUS*-mutant induced pluripotent stem cell (iPSC)-derived MNs (Akiyama et al., 2019).

*Optineurin* (*OPTN*) mutations are implicated in both familial and sporadic ALS (Maruyama et al., 2010). OPTN binds to ubiquitin and regulates NFκB activation and apoptosis (Nakazawa et al., 2016). OPTN is also involved in several selective autophagy processes regulated by TBK1 (Li et al., 2016). Receptor-interacting kinase (RIPK) 1-dependent signaling is suppressed by OPTN through the regulation of its turnover (Ito et al., 2016). OPTN loss leads to progressive demyelination and axonal degeneration through the activation of necroptotic machinery in the central nervous system (CNS) (Ito et al., 2016). These observations suggest that RIPK1 and RIPK3 are significant in the process of progressive axonal degeneration.

A novel variant in *UBQLN4* compromises motor axon morphogenesis in zebrafish, impairing the proteasomal function (Edens et al., 2017; Morrice et al., 2018). Based on these reports, the clearance of junk protein is important in the compartment of the MN axon.

### NMJs

Amyotrophic lateral sclerosis can be redefined as a distal axonopathy disease, because many molecular changes influencing MN degeneration occur at the NMJ (Moloney et al., 2014). The NMJ is a highly specialized synapse, that controls signals between muscles and nerves for skeletal muscle function. Neuromuscular remodeling precedes loss of the motor unit in the mutant *SOD1*-G37R transgenic mouse model (Martineau et al., 2018).

Certain molecules, including galectin-1 (Ferraiuolo et al., 2007; Plachta et al., 2007), CD44 (Schmidt et al., 2011), and amyloid precursor protein (Bryson et al., 2012), affect the function of NMJ. Axon guidance molecules affecting the stability of the cytoskeleton, such as Semaphorin 3A (Venkova et al., 2014), Ephrin A4 (Takata et al., 2013), and Nogo-A (Pradat et al., 2007), have been reported to alter the function of the NMJ in the early stage of ALS. The loss of mitofusin 2 in neurons causes NMJ dysfunction, whereas the upregulation of mitofusin 2 ameliorates the phenotype of mutant *SOD1*-G93A transgenic mice (Wang et al., 2018).

The expression of mutant *FUS* or *FUS* knockdown in zebrafish results in the impairment of motor activity and reduces quantal transmission at NMJs, indicating loss and gain of function of FUS (Armstrong and Drapeau, 2013). These changes in FUS culminate in presynaptic dysfunction at the NMJ (Armstrong and Drapeau, 2013). There is evidence that FUS plays multiple roles in the nucleus and axonal compartments involved in NMJ maintenance and axonal transport (Schoen et al., 2015; So et al., 2018). FUS mediates the regulation of acetylcholine receptor transcription at NMJ and is dysregulated in ALS (Picchiarelli et al., 2019).

C9ORF72 was identified on the presynaptic side where the protein interacts with Rab3 protein family members, suggesting that it has a role in the regulation of synaptic vesicle functions as a guanine nucleotide exchange factor (Frick et al., 2018).

In *Drosophila*, the protein Arc1 is a component of the capsid-like structures that bind *DARC1* mRNA in neurons. These capsids are included in the extracellular vesicles that are transferred across the NMJ from MNs to the muscle cells (Ashley et al., 2018). The transport of mRNA across the NMJ via these retrovirus-like capsids and extracellular vesicles is required for synaptic plasticity (Ashley et al., 2018). Dipeptide repeat proteins related to C9ORF72 spread between cells *in vitro* and *in vivo* (Westergard et al., 2016). Tau is another protein that is transported from donor cells to recipient cells through the cell culture medium (Wu et al., 2016b). Evidence suggests that the mechanism of pathogenic molecular transfer, termed the prion hypothesis, may be activated in the extracellular space and across the NMJ synapses during degeneration of the motor cortex with centrifugal spreading (Furukawa et al., 2011; Nonaka et al., 2013; Porta et al., 2018).

### Aberrant Axonal Branching

Axonal branching is a fundamental mechanism of nervous system neuroplasticity (Menon and Gupton, 2018). Accumulating evidence suggests that aberrant axonal branching is involved in the pathomechanisms of ALS.

Overexpression of mutant human *TARDBP* in zebrafish embryos induces a phenotype that includes shorter MN axons, premature and increased branching, and abnormal swimming (Kabashi et al., 2010). On the other hand, overexpression of progranulin rescues mutant *TARDBP*-induced aberrant axonal branching and short axonal outgrowth (Laird et al., 2010).

Injection of morpholino antisense oligonucleotides to inhibit the translation of target mRNA and to knock down *SMN* in zebrafish embryos significantly increases MN branching (McWhorter et al., 2003). C9ORF72 modulates the activity of the small GTPases, resulting in increased activity of LIM kinases 1 and 2 and regulation of axonal actin dynamics (Sivadasan et al., 2016). Various actin isoforms are expressed in primary mouse MNs, and their transcripts have been observed to be translocated into axons (Moradi et al., 2017). It is proposed that short hairpin RNA-mediated depletion of α-*actin* reduces axonal filopodia dynamics and disrupts collateral branch formation in developing MNs (Moradi et al., 2017).

Temporary overexpression of human cyclin-F (*CCNF)* in zebrafish embryos increases the levels of cleaved caspase-3 and cell death in the spinal cord. The mutant *CCNF* zebrafish also developed an MN axonopathy, which consists of shortened primary MN axons and an increased frequency of aberrant axonal branching (Hogan et al., 2017).

A recent study reported that MNs cultured from mutant *SOD1*-G93A transgenic mouse models exhibit enhanced axonal outgrowth and dendritic branching (Osking et al., 2019). As the level of branching does not correlate with the severity of the disease, in this study, the authors concluded that axonal branching does not affect the disease process. Increased synaptic activity or branching is considered desirable in the field of psychiatric disease (Shao et al., 2019). The authors of the present review identified aberrant axonal branching in *FUS*-mutant iPSC-derived MNs (Akiyama et al., 2019). The sensory axons branching in the presence of nerve growth factor (NGF) can be observed at sites marked by stalled mitochondria. NGF promotes branching through the generation of ATP and active axonal translation of mRNA (Spillane et al., 2013). The mechanism underlying mitochondrial stalling and growth factor distribution in MNs requires examination.

The meaning of axonal branching might be different in each stage of the development (Jung et al., 2012). In the embryonic stage, axon pathfinding and synaptic formation are important. However, in the developed stage, aberrant axon branching might have a disadvantage in terms of normal function of signal transmission. The significance of aberrant axonal branching in the neurodegenerative model *in vivo* has not yet been elucidated.

## Omics Profiling of the Axonal Compartment

The previous section provided an overview of the important pathomechanisms of MN axons. These pathomechanisms have been found to influence each other and cannot be entirely separated. This section reviews the omics analysis of the axon compartment in order to obtain an overall understanding of this complex process occurring in an important region of the neuron.

### Lessons From Different Nervous Systems

The rationale for conducting omics analysis of the axon compartment is as follows (Table 2). Surprisingly complex, constantly changing transcriptomes are present in mature axons. Thus, axonal mRNA localization is likely to be tightly regulated and to play multiple roles. The ribosomal protein S6 has been observed with immunoelectron microscopy in the axons of embryonic sympathetic and hippocampal neurons grown *in vitro* (Tcherkezian et al., 2010), indicating that local mRNA translation also occurs in growing axons. Further, the local translation of proteins from mRNAs selectively transported from the soma to the synaptic terminal appears to be involved in the regulation of axon outgrowth and regeneration (Zheng et al., 2001; Taylor et al., 2009).

**TABLE 2:** Omics analyses of the axon compartment in *in vivo*, *ex vivo*, and *in vitro* models of several types of nervous systems.

Disease modeling	System	Cell resource	*Vivo/Vitro*	Methodology	Analysis	Core result	References
ALS	Motor	Human iPS-derived motor neuron	*in vitro*	Separating axon using microfluidics (Jiksak Bioengineering)	RNA sequencing	Increased level of Fos-B mRNA, the binding target of FUS, in FUS-mutant MNs. While Fos-B reduction using si-RNA or an inhibitor ameliorated the observed aberrant axon branching, Fos-B overexpression resulted in aberrant axon branching even in zebrafish model.	Akiyama et al., 2019
ALS	Motor	Mouse and human stem cell-derived spinal motor axons	*in vitro*	Microfluidics	RNA sequencing	Identified 3,500–5,000 transcripts in mouse and human stem cell-derived spinal motor axons, most of which are required for oxidative energy production and ribogenesis. Axons contained transcription factor mRNAs, e.g., Ybx1, with implications for local functions. In SOD1G93A mutant, identifying 121 ALS-dysregulated transcript, including Nrp1, Dbn1, and Nek1, a known ALS-causing gene.	Nijssen et al., 2018
No	Motor	hiPSC-derived motor neuron	*in vitro*	Permeable inserts culture device	RNA sequencing	Discriminate axonal and somatodendritic compartments	Maciel et al., 2018
No	Retina	Retinal ganglion cells (RGCs) exit from the eye primordia from Xenopus laevis embryos	*Ex vivo*	Axon grow through the 1 μm pores of the transfilter on the Boyden chamber	Pulsed stable isotope labeling of amino acids in cell culture (pSILAC) with ultrasensitive sample preparation technology termed single-pot solid-phase-enhanced sample preparation (SP3)	Axons stimulated by different cues (netrin-1, BDNF, Sema3A) showed distinct signatures with over 100 different nascent protein species	Cagnetta et al., 2018
ALS	Spinal	Dissociated spinal cord culture from ICR mice at E12.5	*Ex vivo*	Modified boyden chamber membrane culture system	RNA sequencing	Elavl2 and miR-146a, miR-126-5p, miR-99a are shared in axons of lentiviral overexpression of both p.A315T TARDBP and p.G93A SOD1 mutants.	Rotem et al., 2017
No	Neuron	Differentiated neurons from human ESC	*in vitro*	Microfluidics	Microarray	Confirmed the presence of two well characterized axonal mRNAs in model organisms, β-actin and GAP43, within hESC-neuron projections. oxytocin mRNA localized to these human projections and confirmed its localization using RNA-FISH.	Bigler et al., 2017
No	Motor	Isolated motor neuron from E12.5 CD-1 mouse spinal cord using p75NTR antibody panning	*Ex vivo*	Xona microfluidics, SND 150 chamber	RNA sequencing	Double-random priming transcriptome methods enable to serially diluted total RNA down to 10 pg	Briese et al., 2016
No	Retina	Retinal ganglion cells (RGCs) of mouse	*in vivo*	Axonal translatome using Axon-TRAP-RiboTag mouse and IP of ribosome mRNAs	*in vivo* axonal translatome	The embryonic to postnatal axonal translatome comprises an evolving subset of enriched genes with axon-specific roles, suggesting distinct steps in axon wiring, such as elongation, pruning, and synaptogenesis. Adult axons have a complex translatome with strong links to axon survival, neurotransmission and neurodegenerative disease.	Shigeoka et al., 2016
SMA	Motor	Isolated motor neuron from E12.5 CD-1 mouse spinal cord using p75NTR antibody panning	*Ex vivo*	Xona microfluidics, SND 150 chamber	Microarray	Knockdown of SMN, the protein deficient in spinal muscular atrophy, produced a large number of transcript alterations in both compartments. Transcripts associated with axon growth and synaptic activity were down-regulated on the axonal side of smn- deficient motor neurons.	Saal et al., 2014
No	Retina	DRG explants dissected from embryonic (E16) and adult (3–5 mo old) from Sprague Dawley rats	*Ex vivo*	Compartmentalized chamber to isolate mRNA from pure embryonic and adult sensory axons devoid of non-neuronal or cell body contamination	Genome-wide microarray	Tubulin-beta3 (Tubb3) mRNA is present only in embryonic axons, with Tubb3 locally synthesized in axons of embryonic, but not adult neurons where it is transported	Gumy et al., 2011
No	Retina	Retinal ganglion cell (RGC) axons of two vertebrate species, mouse and Xenopus	*Ex vivo*	Laser capture microdissection (LCM) to isolate the growth cones	Coupled with unbiased genomewide microarray profiling.	Many presynaptic protein mRNAs are present exclusively in old growth cones. ome receptor transcripts (e.g., EphB4), present exclusively in old growth cones, were equally abundant in young and old cell bodies.	Zivraj et al., 2010
No	Cortical	Cortical and hippocampal dissociated neurons from embryonic Sprague Dawley rats at E18	*Ex vivo*	Microfluidic chamber with microgrooves (7.5 μm wide, 3 μm high)	Microarray	Axonal transcripts are enriched for protein translational machinery, transport, cytoskeleton, and mitochondrial maintenance.	Taylor et al., 2009
No	Motor	Primary DRG cultures from L4-5 were prepared from Sprague Dawley rats that had been injury conditioned 7 days before by sciatic nerve crush at midthigh level	*Ex vivo*	Dissociated DRGs were plated into tissue culture inserts containing porous membranes (8-μm pores). Axons were isolated after 16-20 h in culture by scraping away the cellular content from the upper or lower membrane surfaces	cDNA microarray	Neurotrophins (nerve growth factor, brainderived neurotrophic factor, and neurotrophin-3) regulate axonal mRNA levels and use distinct downstream signals to localize individual mRNAs.	Willis et al., 2007

Elucidation of what features of axonal function require local translation and determination of the mRNAs that mediate these functions have induced intriguing challenges in the field of axonal biology (Deglincerti and Jaffrey, 2012; Jung et al., 2012). Assessment of the axonal transcriptome using microarray studies has identified important axonal mRNAs and has demonstrated the complexity and dynamic nature of the axonal transcriptome (Zivraj et al., 2010; Gumy et al., 2011).

In a pioneering study involving omics analysis in axons, more than 200 different mRNAs were identified with cDNA microarray analysis in axons derived from rat with injured sensory neurons (Willis et al., 2007). Proteins involved in the transcription, synthesis of proteins, intracellular transport, calcium metabolism, mitochondrial functions, and cytoskeletal functions were identified in the study (Willis et al., 2007). The report raised several important questions regarding axonal translation (Deglincerti and Jaffrey, 2012), including the question of why transcripts for nuclear proteins are localized to distal axons.

Using a microfluidic chamber enabling the isolation of axons without contamination with non-axonal material, mRNA has been purified from mature CNS axons (Taylor et al., 2009). The same study also described the localization of catenin-β1 and neurexin-3 mRNA with fluorescence *in situ* hybridization in the axonal compartment (Taylor et al., 2009). The somatodendritic compartments are enriched in transcripts with postsynaptic functions and in nuclear non-coding RNAs such as *7SK*, whereas transcripts related to translation such as*7SL*, the cytoplasmic non-coding RNA, are upregulated in the compartment of the axon fraction (Briese et al., 2016).

Transcriptome-wide analyses have revealed numerous transcripts encoding transmembrane or secreted proteins, which comprise about 13% of the total mRNAs found in growth cones (Zivraj et al., 2010). Transcripts present in axons encode many transmembrane proteins, such as integrins and protocadherins, which are cell adhesion molecules, and EphB4 and Nrp2, which are guidance receptors (Zivraj et al., 2010). Thus, local translation may change the cell adhesion capacity of axons and allow axons to respond to extracellular signaling molecules (Gumy et al., 2011). Axons also contain transcripts that code for secreted proteins, including semaphorin and ephrin, which are guidance molecules; BMP1, CTGF, and FGF, which are growth factors; and collagen and TIMP3, which compose and regulate the extracellular matrix. Thus, it is expected that local translation probably plays a role in the regulation of extracellular components by affecting proteins that are secreted from growth cones (Deglincerti and Jaffrey, 2012). Axons also contain structures that resemble the ER and Golgi. Specific labeling of the ER and Golgi exhibits irregular, punctate staining along the axon, suggesting that axon-specific versions of these organelles may be present in nerve terminals (Merianda et al., 2009).

The development of compartmentalization has enabled the examination of axon pathology in MN diseases. The knockdown of *SMN*, which encodes the protein that is deficient in spinal muscular atrophy (SMA), was shown to produce numerous transcript alterations in both axon and somatic compartments of the microarray (Saal et al., 2014). Transcripts associated with axon growth and synaptic activity are downregulated on the axonal side of *SMN*-deficient MNs. Improvements in the handling of small quantities of RNA have led to further progress in this field (Briese et al., 2016).

Evaluation of cultured spinal cord neurons grown with a compartmented platform and subjected to next-generation sequencing technology revealed that mRNAs and miRNAs are differentially expressed in the somatic compared with the axonal neuronal compartments (Rotem et al., 2017). In axons with lentiviral overexpression of p.A315T *TARDBP* or p.G93A *SOD1* mutants, *Elavl2*, *miR-146a*, *miR-126-5p*, and *miR-99a* are commonly expressed. Examination of the local transcriptome revealed that the most abundant mRNAs within human embryonic stem cell-derived neuronal projections are functionally similar to the rat axonal transcriptome of cortical neurons (Bigler et al., 2017).

The use of microfluidic technology has been particularly useful in neuroscience research. Microfluidic platforms have allowed researchers to address specific questions related to axonal guidance, synapse formation, and cargo transport within axons, and led to the development of three-dimensional (3D) CNS models for pharmacological testing and drug screening (Neto et al., 2016). Human iPSC-derived MNs grown in a culture device with permeable inserts were observed to produce large amounts of enriched axonal material that can be harvested for RNA isolation and sequencing (Maciel et al., 2018). Transcriptome profiling has revealed axonal and somatodendritic compartment-specific expression.

Recently, Nijissen and colleagues developed a refined method named Axon-seq, combining microfluidics, RNA sequencing, and bioinformatics analysis (Nijssen et al., 2018). These results demonstrated that the transcriptome of the axon compartment is quite different from that of the soma and includes a smaller number of mRNAs. They identified up to 5,000 mRNAs in mouse and human stem cell-derived MN axons; the functions of the majority of these are oxidative energy and ribosome production. Axons contain transcription factor mRNAs, implicating local functions. Investigation into the response of degenerated ALS motor axons to the *SOD1*-G93A mutation identified 121 ALS-dysregulated transcripts. Among these, *Nrp1* and *Dbn1* are involved in axonal function, and *Nek1* is a known ALS-causative gene (Brenner et al., 2016; Kenna et al., 2016; Nijssen et al., 2018). Axon-seq is an advanced technique for sequencing the RNA in axons, and thus can provide enhanced knowledge about peripheral nerve biology to explain the vulnerability/resilience of MN (Nijssen et al., 2017; Allodi et al., 2019) and to identify the treatment of MN diseases.

### Development of a Microfluidic Device for Larger-Scale Omics Analysis

Despite the improvement offered by the microfluidic device, harvesting a sufficient volume of lysate from the axon compartment remains challenging. In the process of improving the dimensions of the well and materials, a novel microfluidic device was developed by the authors of this study (Table 3). The device enabled comparison of two sets of isogenic *FUS*-mutant iPSC-derived MNs generated using genome editing technology (Joung and Sander, 2013; Okano and Yamanaka, 2014), and provided observations of increased branching in *FUS*-mutant MN axons compared with those in isogenic controls (Akiyama et al., 2019). This phenotype was confirmed using other ALS-causative mutations, including *SOD1* and *TARDBP*. Combining this innovative microfluidic device (Kawada et al., 2017) with hiPSC-derived MN organoids further revealed the entire *in vitro* profile of the human MN axons. This technique identified increased *Fos-B* mRNA as a binding partner of FUS and as a causative event for aberrant axon morphology both *in vitro* and *in vivo*.

**TABLE 3:** Comparison of our microfluidic devices with those of previous studies.

	Neuron device	Modified boyden	Nerve organoid device
Company	Xona Microfluidics	Corning	Jiksak Bioengineering
Dimension	2 D	2 D	3 D (axon bundle)
Cell type	Primary mouse motor neuron	Primary mouse motor neuron	iPSCs derived motor neuron
Pore size	1∼3 um	3 um	150∼200 um
Axon length	150 um	NA	10,000 μm (1 cm)
Retrievable neurons	∼10*3	5 × 10*5	10*4-
RNA	20 pg-	0.3 ng/μl	12 ng (l ng/μl) -
References	Briese et al., 2016; Bigler et al., 2017; Nijssen et al., 2018, etc.	Rotem et al., 2017; Maciel et al., 2018, etc.	Akiyama et al., 2019; Kawada et al., 2017

Morphological changes in MN axon branching have been found to precede MN death in the mutant *SOD1*-G93A transgenic mouse model (Tian et al., 2016), and abnormal neural branching has been detected in zebrafish that overexpress mutant *FUS* (Armstrong and Drapeau, 2013). Improvements in axon morphology following suppression of abnormally upregulated *Fos-B* in *FUS* mutants suggested a novel therapeutic candidate for *FUS*-mutant ALS.

Previous studies have also reported that upregulation of *Fos-B* mRNA is associated with increases in spines (Lafragette et al., 2017; Cahill et al., 2018) and growth cones (Anastasiadou and Knoll, 2016). δ*Fos-B* modulates immature spines of the nucleus accumbens in a model of drug addiction (Grueter et al., 2013). Certain chemical stimulators such as kainic acid lead to neurodegeneration via upregulated expression of immediate early genes, including that of *Fos-B* (Pereno et al., 2011). The hyperexcitability hypothesis is a major theme in proposing the pathomechanism of ALS (Wainger et al., 2014). A recent report of activator protein-1 (AP-1) and MN degeneration in the mutant *SOD1*-G93A transgenic mouse model has attracted attention (Bhinge et al., 2017). Additionally, the suppression of dual leucine zipper kinase, the upstream signal protein for c-Jun (AP-1 family member), may become a therapeutic target for ALS (Bhinge et al., 2017). Although substantial differences have been reported in *SOD1*-ALS compared with *FUS*-ALS and *TARDBP*-ALS (Fujimori, 2018), *SOD1*-, *TARDBP*-, and *FUS*-mutant MNs have common features, suggesting a role for AP-1 in the neurodegeneration observed in ALS. The Fos-B protein accumulates abnormally in the MNs of ALS patients, including in sporadic cases. Thus, Fos-B appears to be a potential therapeutic target molecule.

The novel microfluidic device described in the preceding paragraph comprises a large canal that enables the collection of sufficient samples of isolated MN axons for RNA sequencing (Kawada et al., 2017). This device has proven useful in visualizing the global profile of the axon compartment. Although other types of microfluidic devices, some of which are specific to cell fraction analysis, are available on the market (Briese et al., 2016; Rotem et al., 2017), they are typically restricted by the limited amount of specimen obtained (Table 3). As only a very small amount of specimen can be analyzed, variation in conditions, such as cell purity and culture procedures, may influence the results. Kawada’s microfluidic device enables analysis with fewer technical biases because it involves the collection of large amounts of macroscopically observable axon bundles. RNA profiles from the axon samples have reproduced the previously reported profiles of the MN axon (Briese et al., 2016; Rotem et al., 2017), justifying the methodology of this novel device. Furthermore, the data obtained may provide important resources for the subcellular fractional analysis of stem cell-derived MN axons.

### Are These mRNAs Translated in Axons?

An important question is whether these mRNAs are translated in axons or transported to the nucleus/cell body. The importance of axonal translation for CNS maintenance is under debate (Spaulding and Burgess, 2017). Several types of mature polarized cells utilize asymmetrical mRNA localization as a means of synaptic communication with other types of cells (Xing and Bassell, 2013). *In vivo*, the longest axons, such as those of mature sensory and motor peripheral neurons, rely most strongly on mRNA transport and local translation to maintain homeostasis.

Upregulation of ribosome synthesis in axons has been found to occur early in the pathogenesis of both mutant *SOD1*-G93A transgenic mouse models and human ALS autopsy samples, which suggests the involvement of Schwann cells in ALS pathology and in aberrant axonal RNA metabolism (Verheijen et al., 2014). Gene expression analyses of the anterior branch of human obturator MNs biopsied from patients with ALS demonstrated upregulation of a cluster of genes that play important roles in biological processes involving RNA processing and protein metabolism (Riva et al., 2016).

Direct evidence for neurodegeneration has been obtained from the observation of mRNA transport dysregulation due to mutations in the RBP SMN1, which causes SMA (Wang et al., 2016b). SMN is present ubiquitously, and its deletion is lethal. However, MNs are more sensitive to SMN reduction than other cell types, possibly because reduced SMN decreases the axonal localization of several mRNAs (Rage et al., 2013) and inhibits the activity of the mammalian target of rapamycin in axons (Kye et al., 2014). An additional role for SMN is in the regulation of axonal localization and local translation of *growth-associated protein 43* (*GAP43*) mRNA in growth cones (Fallini et al., 2016). The overexpression of two mRNA-binding proteins, HuD and IGF2 mRNA-binding protein 1, restores the mRNA and protein levels of GAP43 and has been shown to rescue the axon outgrowth defects in the neurons of an SMA patient (Fallini et al., 2016).

In previous studies, protein interaction screening intended to elucidate *FUS*-mutant phenotypes also identified several molecules that interact with FUS, including SMN (Yamazaki et al., 2012; Groen et al., 2013). Aberrant distribution of SMN in cytosolic FUS accumulations induces SMN reduction in axons. Accumulation of mutant human FUS induces an integrated stress response and reduces protein synthesis in nearby axons (Lopez-Erauskin et al., 2018).

Non-nuclear pools of splicing factor, proline-glutamine rich (SFPQ) are essential for normal motor development via local mRNA maintenance or processing, and the coiled-coil domain of SFPQ is required for axonal localization (Thomas-Jinu et al., 2017). The RBPs modulate nuclear processing, intracellular transport, and local translation of target mRNAs for an accurate spatial and temporal gene expression. SFPQ functions as an RBP because it binds to and modulates numerous neuronal mRNAs, including in cells, such as dorsal root ganglion neurons. SFPQ, which has been identified by subcellular compartmentalization analysis (Cosker et al., 2016; Takeuchi et al., 2018), has been found to orchestrate spatial gene expression, which is essential for axonal viability.

Local translation is also involved in several neurodevelopmental disorders (Batista and Hengst, 2016). Local translation defects are associated with fragile X mental retardation and autism spectrum disorders (Kelleher and Bear, 2008). Fragile X mental retardation protein, which is present in dendritic spines, growth cones, and axons, modulates plasticity (Bassell and Warren, 2008) and the presynaptic proteome (Christie et al., 2009; Akins et al., 2012). In mouse brain slices, loss of the fragile X mental retardation protein was found to perturb the development of presynaptic nerve terminals (Hanson and Madison, 2007).

Degeneration of motor axons results from mutations in various tRNA synthetases, which is consistent with the notion that local translation of transported mRNA is necessary for axonal homeostasis (He et al., 2015; Storkebaum, 2016). Recently, cytoplasmic polyadenylation element-binding protein 4 was found to orchestrate the dysregulation of mRNA expression in autism (Parras et al., 2018). Identification of a master regulator of RNA metabolism would be beneficial in understanding and treating for both diseases that affect MNs and psychiatric diseases.

Nascent chain tracking is a novel technique for visualizing local translation. This method uses multi-epitope tags and antibody-based fluorescent probes to quantify the dynamics of protein synthesis at the level of individual mRNAs (Morisaki et al., 2016). Due to its sensitivity and versatility, nascent chain tracking is a useful tool for quantifying mRNA translation kinetics. Synaptic activity can induce mRNA localization and the local translation of β-actin, which stabilizes expanding synapses at dendritic spines (Wu et al., 2016a; Yoon et al., 2016). Real-time visualization of mRNA translation in the axonal compartment is an innovative method enabling analysis of axonal pathology *in vivo* (Wang et al., 2016a; Yan et al., 2016). The inducible fluorescent probe can be regulated in time and space in neurons and is used to examine the maturation of miRNA. The local maturation of miRNA by synaptic stimulation results in a spatially restricted protein synthesis reduction from the mRNA (Sambandan et al., 2017). The proteomics approach described in a later section adds to the understanding of the global change of nascent proteins produced in the axon fraction.

### Role of Intra-Axonal Transcription Factors

Why transcripts for nuclear proteins are localized to the distal axons is a big question raised by the omics analysis. Fos-B, a mediator of abnormal axonal branching in *FUS*-mutated MNs, is a transcription factor. Another research group also reported dysregulated transcription factors in ALS MNs (Nijssen et al., 2018). In determining the role of transcription factors in the axon compartment, a comprehensive transcription of the action fraction has identified mRNAs encoding a larger amount of transcription factors and co-factors (Ji and Jaffrey, 2014). One example is that of axonal STAT3, which is translated locally, activated upon nerve injury, and is transported retrogradely with dynein and importin α5, modulating the survival of peripheral sensory neurons (Ben-Yaakov et al., 2012). Recently, *Tp53inp2* was reported to be an atypical mRNA regulating axon growth by enhancing the NGF-TrkA pathway independently with translation (Crerar et al., 2019). Importantly, data have indicated that axonal degeneration shared early molecular change in the neurodegenerative process of neurological disorders in aged populations (Dadon-Nachum et al., 2011; Tagliaferro and Burke, 2016; Salvadores et al., 2017).

In the brain of a person with Alzheimer’s disease, inhibition of local translation of *Atf4* mRNA overproduction eliminates amyloid β-induced cell loss (Baleriola et al., 2014; Peng et al., 2016). *Atf4* mRNA translation is controlled by phosphorylation of elongation initiation factor 2a, pivotal for an integrated stress response (Batista and Hengst, 2016). The role of axonal transcription factors in relation to translated proteins and non-translated RNA requires further elucidation.

### Interaction Among the Mechanisms Already Described

The hallmark feature in the majority of autopsy cases of ALS is nuclear depletion and cytoplasmic accumulation of TDP-43 in degenerated neurons (Kim and Taylor, 2017). Thus, dysfunctional trafficking between the nucleus and cytoplasm likely plays a role in the pathology of ALS (Nedelsky and Taylor, 2019) and may also be important in normal physiological aging, Huntington’s disease, and Alzheimer’s disease (Nedelsky and Taylor, 2019). RBPs with prion-like domains (PrLDs) undergo liquid-liquid phase separation to form functional liquids, which can be converted into abnormal hydrogels that contain pathological fibrils that are often seen in neurodegenerative diseases. TDP-43, FUS, heterogeneous nuclear ribonucleoprotein A1 (hnRNPA1), and hnRNPA2 are nuclear RBPs with PrLDs that are incorrectly sent to cytoplasmic inclusions in neurodegenerative diseases. Mutations in PrLDs increase the rate of fibril formation and initiate disease (Guo et al., 2018). Karyopherin-β2, also known as transportin-1, binds the proline-tyrosine NLS and then blocks and reverses FUS, TATA-box-binding protein associated factor (TAF) 15, Ewing sarcoma RBP1 (EWSR1), hnRNPA1, and hnRNPA2 fibril formation. Importin-α and karyopherin-β1 also block and reverse TDP-43 fibril formation. Phase separation, like stress granule formation, is an emerging property of proteins containing PrLD such as FUS (Guo et al., 2018; Hofweber et al., 2018; Qamar et al., 2018; Yoshizawa et al., 2018). T-cell-restricted intracellular antigen-1 (TIA1) mutations were found to delay stress granule disassembly and to promote the accumulation of granules harboring TDP-43 (Mackenzie et al., 2017). *C. elegans* TIAR-2/TIA protein functions cell autonomously to inhibit axon regeneration (Andrusiak et al., 2019). One of the important roles of phase separation is transcription enhancement (Sabari et al., 2018), which might be related to local translation/transcription. The association between axonal dysfunction and these cytoplasmic events, including phase separation, has not yet been elucidated.

There is emerging evidence of interactions among different processes of axonal pathology in ALS. Annexin A11 (ANXA11), a phosphoinositide-binding protein associated with the RNA granule, has the role of a molecular tether between lysosomes and RNA granules. Such tethering is impaired by the ALS-associated *ANXA11* mutation (Smith et al., 2017; Liao et al., 2019). Late endosome bearing mRNA encoding mitochondrial functional molecules stops at mitochondria and these mRNAs are translated on Rab7a endosomes locally in the axon (Cioni et al., 2019).

In summary, exactly how these complex mechanisms are influenced by each other is still unknown. There is a need for understanding how cytoskeletons are maintained, and how molecules are transported/metabolized/synthesized, or abolished when unnecessary. Elucidating the interaction of these mechanisms might answer the vital question of why MNs are vulnerable in ALS.

### Advanced Omics Analysis and Further Consideration

Conducting a comprehensive analysis of the newly produced proteome from limited samples of subcellular compartments that are uncontaminated by the somatodendrite remains a major technical problem (Eichelbaum and Krijgsveld, 2014). Stable isotope labeling of amino acids in cell culture (SILAC) has been combined with single-well solid phase-enhanced sample prep. Using this method, the newly produced proteome of isolated retinal axons was obtained rapidly (in approximately 5 min) (Cagnetta et al., 2018). Treating axons treated with stimuli such as netrin-1, brain-derived neurotropic factor, and Sema3A, has demonstrated distinct proteomes with more than 100 different nascent proteins. Compartment analysis using pulsed SILAC may be applied to ALS cells with a sophisticated culture device.

Using an axon-TRAP-RiboTag approach in mice, the dynamic translatome of axons in the retina *in vivo* matches the subcellular function (Shigeoka et al., 2016). The translatome of the embryonic and postnatal axons includes a changing, enriched set of genes with axon-specific roles. Thus, specific steps in axon wiring, such as axon growth, elimination of unnecessary axons, and synaptogenesis, may be present. Adult axons harbor a complicated translatome that plays a role in axon survival, neurotransmission, and neurodegenerative diseases. Mating of several ALS mouse models can help in precisely understanding mRNA dysregulation. Further transcriptome and proteome analyses using labeled growth cones of single projections in the mouse cerebral cortex *in vivo* may also be of use (Poulopoulos et al., 2019). Spatial transcriptomics is another method for elucidating gene expression in the mouse spinal cord over the disease course, and in postmortem tissue from patients with ALS (Maniatis et al., 2019). Another important approach is the single-cell transcriptomics of nerve organoids *in vitro* (Quadrato et al., 2017); pseudo-time analysis or single-cell trajectory analysis can help establish the relationship between the cause and effect of the transcriptome of the organoids (Xiang et al., 2017; Klaus et al., 2019). Sophisticated neuromuscular co-culture organoids would be beneficial for these studies.

Stimulated Raman scattering microscopy is a new technique for chemical imaging that can be used to map the distribution of various molecules–including lipids, proteins, and nucleic acids–in live cells and tissues, as determined by their intrinsic molecular vibration (Freudiger et al., 2008). The authors of this review used this type of imaging to visualize peripheral degeneration in several ALS mouse models and human postmortem tissue (Tian et al., 2016). Non-labeled live imaging of motor axons may assist in monitoring the time course of axonal pathology *in vivo*.

In clinical settings, the strength-duration time constant, which represents the hyperexcitability of an MN axon, is significantly increased in patients (Kanai et al., 2006, 2012; Geevasinga et al., 2015). Hyperexcitability is thought to be the target of MN death in ALS (Wainger et al., 2014). In cell culture settings, the shortened isoform of TDP-43 is upregulated by neuronal hyperactivation (Weskamp et al., 2019). The role of these short isoform of TDP-43, which might be the product of dysregulation of RNA metabolism, should be considered in the axon fraction. Recent studies have revealed the importance of stathmin-2 (STMN2), a regulator of microtubule stability, in the pathomechanism of *TARDBP* mutation (Klim et al., 2019; Melamed et al., 2019). The expression of a microtubule regulator, *STMN2*, is decreased following *TARDBP* knockdown, when TDP-43 is mis-localized, and in MNs from patients and the spinal cord of postmortem samples. The reduced function of TDP-43 results in the loss of STMN2 due to altered splicing. This is functionally important, as STMN2 is necessary for the outgrowth and regeneration of MN axons. Post-translational STMN2 stabilization rescues neurite outgrowth and axon regeneration deficits by TDP-43 depletion (Klim et al., 2019). A reduction in TDP-43 inhibits axonal regeneration of iPSC-derived MNs, whereas rescue of the expression of STMN2 restores the axonal regeneration capacity (Melamed et al., 2019). The effect of the short form of TDP-43 or cryptic exons under the control of TDP-43 (Ling et al., 2015) should be examined in the axon fraction.

## Concluding Remarks

As described in the preceding section, advanced omics approaches, *in vivo* analysis, and axon–cytoplasmic interactions should be examined as the next steps in investigating axonal pathology in neurodegenerative disease research. The novel concept of microfluidic devices, including the nerve organoid device presented by the authors of this review, should be applied to other neuron types, co-culture systems, or proteomics analyses using human pluripotent cells, because this technique may help elucidate the resilience of long MN and the pathomechanism of ALS.

## Author Contributions

NS, TA, and MA prepared the manuscript. All authors read, revised, and approved the final version of the manuscript.

## Conflict of Interest

The authors declare that the research was conducted in the absence of any commercial or financial relationships that could be construed as a potential conflict of interest. The reviewer MK declared a past co-authorship with the authors NS and MA to the handling Editor.
